# Transcriptional Biomarkers in Oral Cancer: An Integrative Analysis and the Cancer Genome Atlas Validation

**DOI:** 10.31557/APJCP.2021.22.2.371

**Published:** 2021-02

**Authors:** Kinjal D Patel, Hemangini H Vora, Prabhudas S Patel

**Affiliations:** *The Gujarat Cancer & Research Institute, Civil Hospital Campus, Asarwa, Ahmedabad-380 016, Gujarat, India. *

**Keywords:** Biomarkers, expression profiling by array, integrative analysis, oral cancer, the cancer genome atlas

## Abstract

**Objective:**

An impervious mortality rate in oral cancer (OC) to a certain extent explains the exigencies of precise biomarkers. Therefore, the study was intended to identify OC candidate biomarkers using samples of healthy normal tissues (N=335), adjacent normal tissues (N=93) and OC tissues (N=533) from online microarray data.

**Methods:**

Differentially expressed genes (DEGs) were recognised through GeneSpring software (Fold change >4.0 and ‘p’ value <0.001 with Benjamini Hochberg false discovery rate). The DEGs were analysed for their functional annotation and network using GeneCodis 4.0 and STRING databases. The DEGs were further cross-examined in the cancer genome atlas (TCGA) database.

**Results:**

197, 229 and 104 DEGs were spotted from three comparisons: i) OC tissues against healthy normal tissues, ii) OC tissues against adjacent normal tissues and iii) adjacent normal tissues against healthy normal tissues, respectively. Functional gene set enrichment unveiled significance of focal adhesion pathway in OC initiation and progression. Advanced analysis of TCGA cohort (n=345) recognised 85 genes that were altered in ≥10% OC patients for mutations, copy number variations, m-RNA expression and protein expression. Strikingly, the elected 5-gene panel (YWHAZ, RHOA, DLG1, LY6E and PLEC) showed the location on chromosome 3 and 8 and each gene was found to be altered in ≥20% OC patients for m-RNA expression. Further, TCGA validation demonstrated significant association of EIF4A2, CTNNA1 and PMEPA1 expression with overall and disease-free survival.

**Conclusion:**

Using this integrative approach, our study identified prominent transcriptional biomarkers which may be significant targets for OC therapy. Additional validation of these biomarkers in experimental prospective and retrospective studies will launch them in OC clinics.

## Introduction

The rising global incidence of oral cancer (OC) and unaffected OC survival over past few decades compel better approaches to detect early, enhance treatment choices and foretell outcome (Bray et al., 2018; Torre et al., 2015). Gene expression profiling, one of the modern advances is being employed in cancer research as it offers assistance in understanding the disease mechanisms and treatment resistance (Uhlen et al., 2017). These attempts in prostate cancer, breast cancer and ovarian cancer have expedited the development and implementation of gene expression profiling tests such as Mamma print and Oncotype Dx in clinics (Ademuyiwa et al., 2011; Madden et al., 2013). Nevertheless, the establishment of OC biomarkers for use in diagnosis and prognosis has remained obscure.

A few gene expression profiling studies have been carried out in OC that enumerates potential biomarkers of disease identification, progression and response to treatment (Chen et al., 2008; Eslami et al., 2015; Lohavanichbutr et al., 2013; Patel et al., 2020; Ye et al., 2008). Even so, it is strenuous to find the same set of genes as OC biomarkers in published literature; thus far, these biomarkers have not been applied in clinical settings. The foremost troubles in biomarker identification using high throughput technologies are their selection and validation as it provides a huge amount of data. Further, there is a considerable discrepancy in the controls for gene expression profiling. Generally, histologically normal tissue adjacent to the tumor used for the comparison with the postulation that histologically normalcy infers biological normalcy (Farah et al., 2016; Lohavanichbutr et al., 2013; Ye et al., 2008). Alas, it was proven erroneous through the concept of “field cancerization” (Mohan and Jagannathan, 2014). The troubles are also augmented in view of limited sample size and considerable discordance between gene expression profiling studies as to microarray platforms (eg. Affymetrix, Agilent, Illumina), gene chip model (eg. Affymetrix HU-U133A, HU-U95A), sampling process and experimental process (Li and Wong, 2001). 

The public microarray data archives like gene expression omnibus (GEO) and array express along with advanced computational data analysis tools such as GeneSpring GX, INMEX and R/Bioconductor allow us to congregate and re-examine gene expression data in a single experiment. Ergo, various groups have effectively extracted significant insights using meticulous analytical strategies (Liu et al., 2018; Reddy et al., 2016; Zhao and Li, 2018). Here, to overcome the aforementioned limitations, we integrated single chip microarray data (HG-U133_Plus_2) and stratified normal samples into two classes: healthy normal tissues and adjacent normal tissues. The analysis was carried out to identify differentially expressed genes (DEGs) and biological pathways among different groups compared. We also validated the catalogue of DEGs with the cancer genome atlas (TCGA) data for alterations, disease-free survival (DFS) and overall survival (OS) in OC cohort to verify the findings of the integrative analysis. Our attempts in turn recorded the repertoire of OC associated biomarkers which can be used for the disease management.

## Materials and Methods

**Figure 1:**
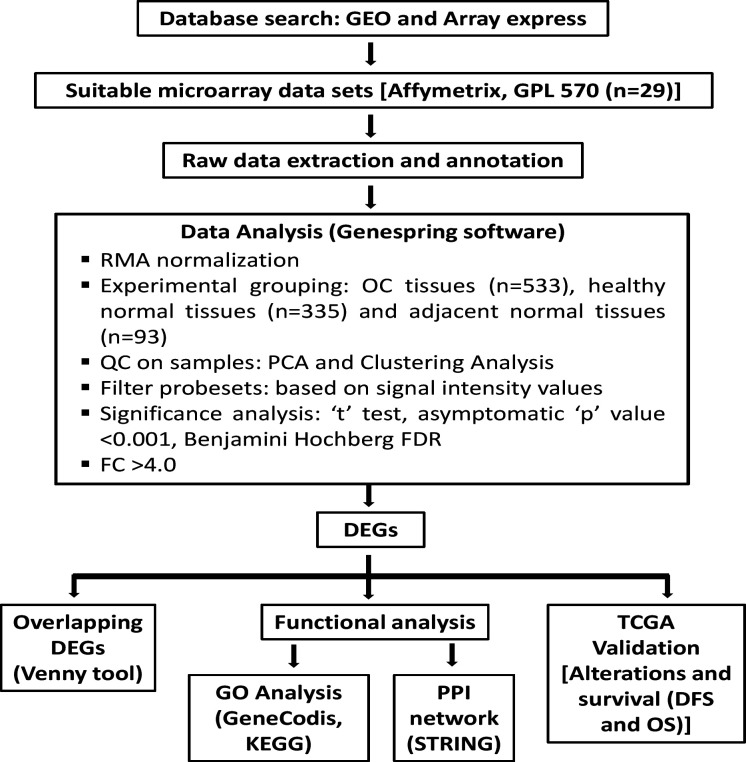
The Basic Work-Flow Demonstrating the Integrative Analysis Study Design. DEGs, differentially expressed genes; DFS, disease-free survival; FC, fold change; FDR, false discovery rate; GEO, gene expression omnibus; GO, gene ontology; KEGG, kyoto encyclopedia of genes and genomes; OC, oral cancer; OS, overall survival; PCA, principal component analysis; PPI, protein-protein interaction; QC, quality control; RMA, robust multi-array analysis; TCGA, the cancer genome atlas

The basic workflow was followed stepwise to execute the integrative analysis as illustrated in Figure 1. The comprehensive procedure was as follows.

*Database search and data mining*

Gene expression profiling datasets for OC were identified through screening of the publicly available datasets, GEO (NCBI) [http://www.ncbi.nlm.nih.gov/geo] and array express (EBI) [http://www.ebi.ac.uk/arrayexpress] using the following keywords in different blends: ‘oral’, ‘head and neck’, ‘cancer’, ‘carcinoma’ ‘homo sapiens’ and ‘expression profiling by array’ until December, 2019. The criteria to select eligible datasets to create the meta-dataset were as follows: i) studies carried out only on human samples were included, ii) studies of head and neck cancer in which the details of sub-sites are obtainable were considered and only samples of oral cavity sites were taken, iii) studies/samples including healthy and unaffected oral sites were incorporated, iv) studies/samples with other oral conditions, such as epithelial dysplasia and/or premalignant lesions were eliminated and v) samples with no source details were kept out of the analysis. All along, it was noted that maximum numbers of eligible studies were of Affymetrix HG-U133_Plus_2 array (GPL 570), hence, the integrative analysis was focused on the single microarray chip model.

*The integrative analysis using GeneSpring software*

The accession number, sample type, number of samples, references and raw data were retrieved for each study. The assessment was performed using GeneSpring software v14.9.1 (Agilent, California, USA). The data was baseline transformed and normalised by robust multi-array analysis (RMA). The data files were further categorized as ‘OC tissues’, ‘healthy normal tissues’ and ‘adjacent normal tissues’. Clustering of each group was checked using principal component analysis (PCA) in order to examine clear stratification between the classified groups. Gene entities were filtered with regard to signal intensity values. ‘t’ test was used for comparison between: i) OC tissues and healthy normal tissues, ii) OC tissues and adjacent normal tissues and iii) adjacent normal tissues and healthy normal tissues. The ‘p’ value computation was asymptotic and Benjamini Hochberg false discovery rate (FDR) was applied to get corrected ‘p’ value. The DEGs were selected based on ‘p’ value (<0.001) and fold change (FC>4.0). The Entrez id, gene symbol and other gene descriptions were exported to Microsoft excel. Gene ontology (GO) analysis was also carried out by integral tools in GeneSpring software. The overlapping DEGs among the groups compared were derived by drawing Venn diagrams using venny v2.1.0 tool (https://bioinfogp.cnb.csic.es/tools/venny/). 

*Functional annotation*

The DEGs were introduced to online bioinformatics tool, GeneCodis 4.0 (http://genecodis.genyo.es/) (Tabas-Madrid et al., 2012) to uncover molecular function of genes and annotate cellular localization of gene products. The pathway enrichment analysis was also done based on the kyoto encyclopedia of genes and genomes (KEGG) database. 

*Interaction network construction*

The network was constructed to envisage direct (physical) and/or indirect (functional) protein-protein interactions (PPIs) across the DEGs and analysed to identify the potential hub proteins for OC using the STRING database (STRING v11.0) (http://string-db.org/) (Szklarczyk et al., 2017). The interaction maps were derived from integrated interaction data that were gained from the four sources, namely previous knowledge, conserved co-expression, genomic context and high throughput experimentation. 

*Virtual validation using TCGA database*

The virtual OC cohort (n=345) was created in TCGA database (http://www.cbioportal.org) (Cerami et al., 2012). The catalogue of DEGs was compared in the cohort for their mutation, copy number variation (CNV), m-RNA expression and protein expression data. The markers with high alterations were selected. The genes were further checked for survival data (DFS and OS) in association with their m-RNA expression values to evaluate their clinical implications.

## Results

*Datasets included in the meta-dataset*

After electronic search, data mining identified Affymetrix platform, Gene chip model: U133 plus 2.0 (GPL570) as a most commonly used technology for transcriptome scrutiny. Based on the inclusion and exclusion criteria, 29 data sets were enrolled in the study. Overlapped samples in datasets were considered only once in the meta-dataset. Thus, overall, the meta-dataset comprised total 961 samples: 533 OC tissues, 335 healthy normal tissues and 93 adjacent normal tissues. The details of the selected datasets are shown in Supporting File 1. The sub-sites of OC malignant and normal tissues were buccal mucosa, tongue, gingiva, floor of mouth and hard palate. 

*Differentially expressed genes in the meta-dataset*

The data were analysed as a single experiment in GeneSpring software. All three groups displayed distinguished expression patterns in the PCA plot. This implies that all the samples were good to fit for further analysis. A total of 54,675 probes were profiled in all the samples. The default analysis setting (‘p’ value <0.05 with Benjamini Hochberg FDR and FC >2.0) identified more than 1700 gene entities in each group compared. Therefore, the criteria of at-least 4 FC and ‘p’ value <0.001 with Benjamini Hochberg FDR was used to further narrow down on DEGs which were both biologically and statistically highly significant. The integrated analysis of OC tissues against healthy normal tissues gave a list of 197 DEGs, of which 98 were up-regulated and 99 were down-regulated. When expression mapping of OC tissues was compared with adjacent normal tissues, a total of 229 DEGs, 134 up-regulated genes and 95 down-regulated genes were detected. Interestingly, in agreement with our deduction, we also identified significant dissimilarity in expression patterns between adjacent normal tissues and healthy normal tissues; total 104 genes were dysregulated in which predominantly 102 genes showed lower expression in adjacent normal tissues than healthy normal tissues. The lists of DEGs for each comparison are presented in Supporting File 2. 

*Gene ontology and pathway enrichment for differentially expressed genes*

To get insights into the functions of obtained DEGs, we performed an enrichment analysis using web-based software, GeneCodis 4.0. The significantly enriched GO terms in biological process, molecular functions and cellular compartments were enlisted in Supporting File 3. For DEGs in OC tissues against healthy normal tissues, the most significant KEGG pathways enriched were viral protein interaction with cytokine and cytokine receptor (hsa04061; p=6.52E-06), IL-17 signaling (hsa04657; p=7.17E-06) and ECM-receptor interaction (hsa04512; p=0.000192). The analysis with regard to DEGs in OC tissues against adjacent normal tissues, indicated that in KEGG pathways, ECM-receptor interaction (hsa04512; p=2.04E-07), protein digestion and absorption (hsa04974; p= 2.51E-07) and IL-17 signaling (hsa04657; p=1.82E-06) showed significant enrichment. It was found that for DEGs in adjacent normal tissues against healthy normal tissues, the significantly augmented terms for KEGG pathways were salmonella infection (hsa05132; p=0.0031677), bacterial invasion of epithelial cells (hsa05100; p=0.00546264) and adherens junction (hsa04520; p=0.00598445) (Table 1).

*Interaction network construction*

**Figure 2:**
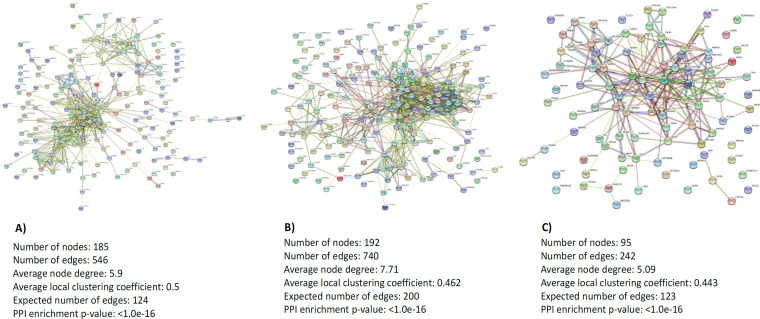
The Protein-Protein Interaction Network for Differentially Expressed Genes in Each Compared Groups: a. oral cancer tissues vs healthy normal tissues; b, oral cancer tissues vs adjacent normal tissues; and c, adjacent normal tissues vs healthy normal tissues

The PPI network was constructed using the list of DEGs for each compared groups in the STRING database which is illustrated in Figure 2. The database analysis regarding OC tissues and healthy normal tissues recognised 185 nodes and 546 edges. The significant hub proteins contained FN1 (node=39), MMP9 (node=37), CXCL8 (node=34) and CXCL10 (node=31). The PPI network of dysregulated genes in OC tissues than adjacent normal tissues showed 192 nodes, and 740 edges. FN1 (node=59), MMP9 (node=45), COL1A1 (node=35) and COL1A2 (node=34) were significant hub proteins in this network. Total 95 nodes and 242 edges were observed in the established network for adjacent normal tissues and healthy normal tissues with YWHAZ (node=21), CDH1 (node=17), CFL1 (node=16) and ENO1 (node=16) as significant hub proteins.

*Meta-gene signatures among different groups compared*

**Figure 3:**
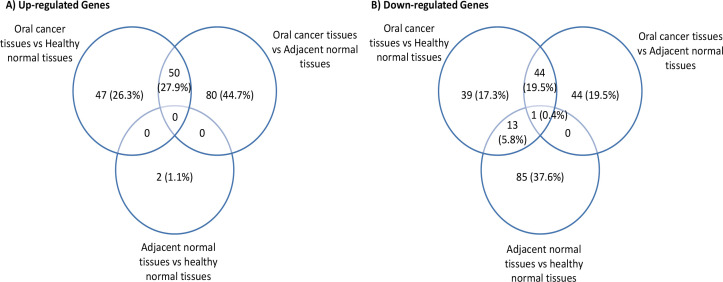
The Overlapping Differentially Expressed Genes three compared groups. a, up-regulated genes; b, down-regulated genes

The overlapping DEGs of three compared groups were identified by drawing Venn diagrams and shown in Figure 3. The comparison revealed 94 genes among which 50 genes were up-regulated and 44 genes were down-regulated in OC tissues as compared to both normal tissues (healthy normal and adjacent normal). Total 47 and 80 genes were found to be significantly elevated in OC tissues only against healthy normal tissues and adjacent normal tissues, respectively. Similarly, 39 and 44 genes displayed significant low expressions in OC tissues only against healthy normal tissues and adjacent normal tissues, respectively. The expressions of 2 and 85 genes were significantly higher and lower in adjacent normal tissues than healthy normal tissues, respectively. As 13 genes were expressed a little in OC tissues and adjacent normal tissues in comparison of healthy tissues, but displayed no significant expression difference between OC tissues and adjacent normal tissues. Merely, 1 down-regulated gene was noted as a common gene entity in all three compared groups (Supporting File 4). 

*Cross-examination with the cancer genome atlas data*

The DEGs in the meta-dataset were interrogated in TCGA database to find mutations, CNVs, m-RNA alterations and protein alterations in OC cohort (N=345). A total of 85 genes were altered in at least 10% of the patients, while 8-gene signatures, LY6K, LY6E, TP63, PLEC, SKIL, YWHAZ, DLG1 and RHOA were altered in more than 25% of samples for mutations, CNVs, m-RNA expressions and protein expressions (Supporting File 5). The top genes based on the m-RNA alteration percentages in TCGA are enlisted in Table 2. Five genes: YWHAZ, RHOA, DLG1, LY6E and PLEC showed gene expression alterations in at-least 20% of OC samples. 

**Figure 4:**
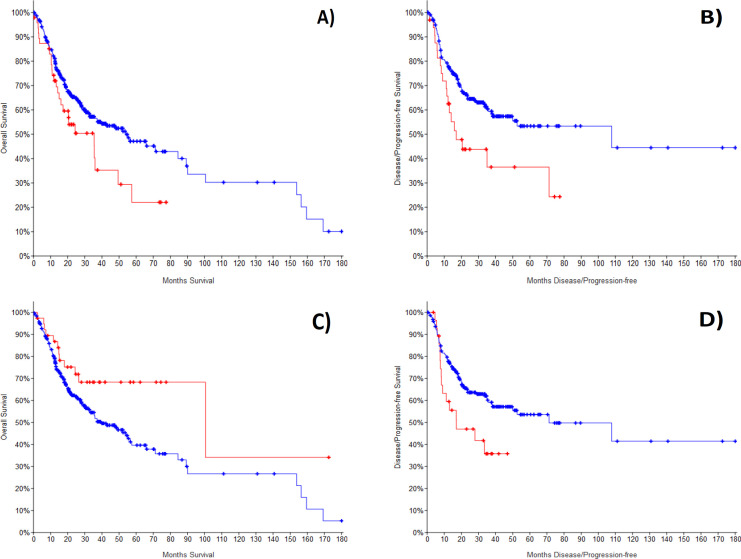
Survival Analysis of Selected Makers in the Cancer Genome Atlas Database. (a), overall survival for EIF4A2 (p=0.0364); (b), disease free survival for EIF4A2 (p=0.0107); (c), overall survival for PMEPA1 (p=0.0337); (d), disease free survival for CTNNA1 (p=0.0232).

Among these genes, at m-RNA expression levels, only EIF4A2 showed significant association with OS and DFS in TCGA database (Figure 4 a-b). The 47 cases with higher EIF4A2 expression demonstrated poor survival as compared to the 295 cases without alterations (35.45 vs 53.91 months; p=0.0364). Similarly, the 32 cases with up-regulation of EIF4A2 showed worse treatment outcomes as compared to the 219 cases without alterations (16.98 vs 107.82 months; p=0.0107). In addition, altered PMEPA1 expression was significantly associated with longer OS (100.49 vs 39.49; p=0.0337), whereas altered CTNNA1 expression was significantly associated with shorter DFS (17.05 vs 71.22; p=0.0232) (Figure 4 c-d).

**Table 1:** Top 15 KEGG Pathways for Differentially Expressed Genes

No.	Pathway ID	KEGG Term	No. of Genes	Corrected ‘p’ Value
OC tissues vs. Healthy normal tissues				
1	hsa04061	Viral protein interaction with cytokine and cytokine receptor	10	6.52E-06
2	hsa04657	IL-17 signaling pathway	10	7.17E-06
3	hsa04512	ECM-receptor interaction	8	0.000192
4	hsa04060	Cytokine-cytokine receptor interaction	13	0.000358
5	hsa05146	Amoebiasis	8	0.000436
6	hsa04062	Chemokine signaling pathway	10	0.000735
7	hsa00350	Tyrosine metabolism	5	0.000773
8	hsa04668	TNF signaling pathway	7	0.003381
9	hsa05204	Chemical carcinogenesis	6	0.003796
10	hsa05222	Small cell lung cancer	6	0.006394
11	hsa04620	Toll-like receptor signaling pathway	6	0.008784
12	hsa05165	Human papillomavirus infection	11	0.008829
13	hsa04933	AGE-RAGE signaling pathway in diabetic complications	6	0.009084
14	hsa05202	Transcriptional misregulation in cancer	8	0.009088
15	hsa05219	Bladder cancer	4	0.009202
OC tissues vs. Adjacent normal tissues				
1	hsa04512	ECM-receptor interaction	12	2.04E-07
2	hsa04974	Protein digestion and absorption	12	2.51E-07
3	hsa04657	IL-17 signaling pathway	11	1.82E-06
4	hsa04510	Focal adhesion	14	1.12E-05
5	hsa05146	Amoebiasis	10	2.60E-05
6	hsa04933	AGE-RAGE signaling pathway in diabetic complications	9	0.000141
7	hsa05165	Human papillomavirus infection	16	0.000146
8	hsa04151	PI3K-Akt signaling pathway	15	0.001095
9	hsa04926	Relaxin signaling pathway	8	0.005343
10	hsa04061	Viral protein interaction with cytokine and cytokine receptor	7	0.005663
11	hsa04610	Complement and coagulation cascades	6	0.013473
12	hsa05323	Rheumatoid arthritis	6	0.016973
13	hsa05222	Small cell lung cancer	6	0.017279
14	hsa00350	Tyrosine metabolism	4	0.017942
15	hsa05219	Bladder cancer	4	0.023482
Adjacent normal tissues vs. Healthy normal tissues^†^				
1	hsa05132	Salmonella infection	9	0.0031677
2	hsa05100	Bacterial invasion of epithelial cells	5	0.00546264
3	hsa04520	Adherens junction	5	0.00598445
4	hsa04670	Leukocyte transendothelial migration	6	0.00773211
5	hsa04390	Hippo signaling pathway	7	0.0100045
6	hsa04145	Phagosome	6	0.0171834
7	hsa04015	Rap1 signaling pathway	7	0.0196547
8	hsa00010	Glycolysis / Gluconeogenesis	4	0.0251694
9	hsa04530	Tight junction	6	0.0261049
10	hsa05135	Yersinia infection	5	0.0267642
11	hsa05418	Fluid shear stress and atherosclerosis	5	0.0391383
12	hsa05203	Viral carcinogenesis	6	0.041833
13	hsa04510	Focal adhesion	6	0.0434251
14	hsa04810	Regulation of actin cytoskeleton	6	0.0477088

^†^Only 14 terms were significantly enriched for KEGG pathway. Abbreviations: KEGG, kyoto encyclopedia of genes and genomes; OC, oral cancer.

**Table 2:** List of Genes Based on the Percentage Gene Expression Alterations in the Cancer Genome Atlas Data

		Chromosome Location	Alteration in m-RNA expression (N=345)	
No.	Gene Symbol		No. of Cases (Altered/Profiled)	Percentage
1	*YWHAZ*	chr8q23.1	98/339	29%
2	*RHOA*	chr3p21.3	90/339	27%
3	*DLG1*	chr3q29	81/339	24%
4	*LY6E*	chr8q24.3	77/339	23%
5	*PLEC*	chr8q24	69/339	20%
6	*LY6K*	chr8q24.3	63/339	18%
7	*TP63*	chr3q28	63/339	18%
8	*FSCN1*	chr7p22	56/339	17%
9	*SKIL*	chr3q26	58/339	17%
10	*ATP6V1A*	chr3q13.31	54/339	16%
11	*RAB6A*	chr11q13.3	51/339	15%
12	*WDR1*	chr4p16.1	51/339	15%
13	*CFL1*	chr11q13	48/339	14%
14	*EXOC5*	chr14q22.3	49/339	14%
15	*EIF4A2*	chr3q28	47/339	14%
16	*PICALM*	chr11q14	45/339	13%
17	*PHLDB2*	chr3q13.2	39/339	12%
18	*LAPTM4B*	chr8q22.1	41/339	12%
19	*CTNNA1*	chr5q31.2	40/339	12%
20	*PMEPA1*	chr20q13.31-q13.33	38/339	11%
21	*HRASLS*	chr3q29	36/339	11%
22	*GPBP1L1*	chr1p34.1	38/339	11%
23	*SERPINH1*	chr11q13.5	35/339	10%
24	*STIP1*	chr11q13	35/339	10%
25	*MAGEA3*	chrXq28	33/339	10%
26	*VAMP3*	chr1p36.23	33/339	10%
27	*ACTR2*	chr2p14	33/339	10%

## Discussion

The use of high throughput global transcriptome profiling for molecular characterization of cancers appears to be a credible approach to discover candidate cancer biomarkers. Nonetheless, the clinical utilization of data is extremely challenging by reason of data analysis, discordance among studies and biomarker validation. The discordance may be either deliberate to biological heterogeneity or technical artefacts. These facets lay emphasis on integrative analysis, a systemic method that analyse individual experiments as single to increase the power, develop a more correct estimate of effect magnitude and resolve ‘the uncertainty. Hence, the prime objective of the present study was to recognise clinically relevant biomarkers for OC employing this statistical procedure.

Adopting a similar method, reports on microarray datasets have led the discovery of novel biomarkers in thyroid cancer, gastric cancer and head and neck cancer (Liu et al., 2018; Reddy et al., 2016; Zhao and Li, 2018). This is also translated in clinics to predict recurrence and treatment outcome in breast cancer (Ademuyiwa et al., 2011; Madden et al., 2013). Earlier investigations also have analysed public repositories on gene expression profiling for OC. Two such studies by Osathanon et al., (2016) and Sun et al., (2016) identified important molecular contributors in OC development. Another study by Reis et al., (2011) identified 4-gene signature in histologically normal surgical margins to predict recurrence through testing 5 GEO datasets along with their own dataset. However, none of these studies included microarray experiments which were aimed to characterize normal oral tissues. Additionally, in the recent analysis, Makarov and Gorlin, (2019) have incorporated only healthy normal buccal mucosa samples of single dataset (GSE17913) to obtain a gene set for OC diagnosis. Our study integrated suitable samples from such experiments, restricted the analysis on a single platform and categorised controls as per their sources. These not only enhanced the study power, but also avoided cancer/healthy mosaicity and checked the degree of difference in their expression profiles. Further, validation using TCGA data imparted higher assurance rate to selected biomarkers in terms of their clinical use.

Our study noted substantial differences between adjacent normal tissues and healthy normal tissues in the expression patterns, thereby defining molecular divergence among them. This is in accord with a comprehensive analysis of genotype-tissue expression project and TCGA transcriptome data (Aran et al., 2017). The authors indicated that histologically normal tissue adjacent to the tumor presents a unique intermediate state between healthy and tumor. Moreover, other studies have observed that altered biomarkers in adjacent normal tissues can predict cancer recurrence and disease prognosis (Kuan et al., 2015; Reis et al., 2011). Altogether, these are suggestive of penalties due to presence of molecular alterations in adjacent normal tissues which are also found in primary tumors. Such molecular changes consequently, may designate premalignant or malignant clones which were left behind even after surgical removal and may be involved in tumor progression and treatment relapse (Reis et al., 2011). Thus, to better understand this phenomenon, DEGs were subjected to GO and pathway analysis that revealed focal adhesion (hsg04510) as a common cancer related pathway in all three groups compared. This particular observation further strengthens our premise and confirmed that comparison among OC tissues, healthy normal tissues and adjacent normal tissues is a valuable approach in spotting DEGs and pathways associated with OC initiation and progression.

Herein, a 5-gene signature (YWHAZ, RHOA, DLG1, LY6E and PLEC) was elected for OC using an integrative analysis of 29 microarray datasets and validation using a RNA sequencing TCGA dataset. The signature is based on the genes that were dysregulated in any of the comparisons in meta-cohort and showed high m-RNA alterations in TCGA cohort. Of great interest, these genes are found to be located on different regions of chromosome 8 (3/5) and chromosome 3 (2/5). Besides, the majority of DEGs which showed high m-RNA alterations in TCGA cohort showed the location on chromosome 3, 8 and 11 regions (Table 2). Surprisingly, alterations in the same chromosomes have been recurrently identified in OC (Ambatipudi et al., 2011; Garnis et al., 2004; Vincent-Chong et al., 2017). Genomic profiling studies have reported frequent amplifications/gains in multiple chromosomal regions involving 3q, 8q and 11q in OC cell lines and tissue samples (Ambatipudi et al., 2011; Garnis et al., 2004; Martin et al., 2008; Vincent-Chong et al., 2017). Vincent-Chong et al., (2017) have also demonstrated significant relations of 8q and 11q amplification with histological parameters, such as tumor size, pathological staging and lymph node metastasis. The data of Garnis et al., (2004) indicated the potential role of altered genes on 8q in oral premalignant lesions progression towards OC. Further, Martin et al., (2008) have suggested that 11q13 amplification may offer prognostic value in the OC management. Along with other documented studies, our results imply that genes located on these regions might have key tasks in oral carcinogenesis.

Among 5 genes, YWHAZ is the most frequently altered in OC patients of TCGA dataset. This 14-3-3 family protein, when up-regulated, can activate many signalling pathways influencing cell cycle, cell growth, migration/invasion and apoptosis and therefore acts as an oncogene in several cancer types (Gan et al., 2020). A study by Han et al., (2015) has further found over-expression of YWHAZ and verified its involvement in tumor inflammation and immune response through Stat3 signaling in OC. Likewise, RHOA, the second most frequently altered gene in OC patients of TCGA dataset encodes small GTPase protein that participates in the regulation of the intracellular signal transduction (Karlsson et al., 2009). It has been proven that RHOA up-regulation is tightly connected with cancer progression, treatments and prognosis (Song et al., 2017). Yan et al., (2014) have shown that RHOA silencing hinders cell proliferation via cell cycle regulation and migration/invasion via Wnt/β catenin pathway in tongue cancer. Further, as per Huaitong et al., (2017) and Zainal et al., (2018), RHOA has pivotal roles in OC cell proliferation, motility and angiogenesis. In our evaluation, DLG1 and LY6E displayed almost similar alterations frequency in the validation set. DLG1 belongs to membrane associated guanylate kinases family with functions in cell adhesion, tight junction and cell polarity (Marziali et al., 2019), whereas LY6E is a member of the lymphostromal cell membrane Ly6 superfamily with functions in cell adhesion and T cell development (Lv et al., 2018). Numerous studies have documented that their altered expression promotes growth, progression, metastasis and drug resistance in different cancers (AlHossiny et al., 2016; Lv et al., 2018; Zhu et al., 2017). Yet, their biological purpose and clinical importance in human OC are not largely known. Lastly, PLEC, a cytoskeleton linker protein exhibited alterations in 20% OC samples of TCGA cohort. An in-vitro study by Chaudhari et al., (2017) has revealed that PLEC regulates cell motility, invasion and tumorigenicity in OC cells. Katada et al., (2012) and Rikardsen et al., (2015) have as well reported association of high PLEC in OC tumors with worse treatment outcome. So, detection of aforesaid genes in cross-validation analysis demands future in-depth studies to use them as OC biomarkers and/or therapeutic targets.

With more clinical relevance, the study outcome clearly stated prognostic utility of EIF4A2, CTNNA1 and PMEPA1 in OC. These genes were frequently altered in OC samples of TCGA cohort and exhibited significant association with DFS and/or OS. EIF4A2, a eukaryotic translational initiation factor takes part in protein synthesis that might be related to an altered translational landscape of cancer cells (Raza et al., 2015). TCGA data for other cancers and experimental data of Chen et al., (2019) have established high EIF4A2 as a poor prognostic indicator in a variety of cancers. Further, they also suggested that high EIF4A2 promotes metastasis and drug resistance. CTNNA1 encodes catenin alpha 1 protein, which is an important regulator of intracellular adhesion (Sakaki et al., 1999). It is experimentally confirmed that altered expression of CTNNA1 is correlated with dysfunction in E-cadherin mediated cell adhesion in cancer cells and also grants metastatic abilities (Tanaka et al., 2003). Accordingly, Chow et al., (2001) and Tanaka et al., (2003) have found low CTNNA1 expression in OC patients who showed LN metastasis and recurrent tumors. PMEPA1 is a transmembrane protein and controls negative feedback loops in androgen receptor and TGFβ signalling (Itoh and Itoh, 2018). The over-expression of this gene was demonstrated in various cancers excluding prostate cancer. Studies have also reported that altered PMEPA1 expression accelerates cancer cell growth and metastasis and therefore influences disease prognosis (Itoh and Itoh, 2018; Xu et al., 2017). At last, further large scale studies in diverse cohorts are necessary to illuminate the clinical applicability of these prognostic markers in OC. 

In conclusions, the study combining GEO, array express, the integrative analysis and TCGA registered a catalogue of eminent OC biomarkers with high reliability rate in respect to their biological and clinical significance. The elected 5-gene signature, YWHAZ, RHOA, DLG1, LY6E and PLEC demonstrated high m-RNA alterations in OC. The survival data established that EIF4A2, CTNNA1 and PMEPA1 expressions were closely associated with poor DFS and/or OS. However, patient based validation was not performed in the study. Indeed, to draw more critical conclusions about their diagnostic, prognostic and therapeutic utility, large retrospective and prospective clinical studies are needed.

*Supporting Information*

Supporting File 1. Details of data sets included in the meta-dataset (XLSX)

Supporting File 2. List of up-regulated and down-regulated gene entities in (a) oral cancer tissues than healthy normal tissues (b) oral cancer tissues than adjacent normal tissues and (c) adjacent normal tissues than healthy normal tissues (XLSX)

Supporting File 3. The significantly enriched gene ontology terms (top 15) for differentially expressed genes in (a) oral cancer tissues than healthy normal tissues (b) oral cancer tissues than adjacent normal tissues and (c) adjacent normal tissues than healthy normal tissues (XLSX)

Supporting File 4. The list of (a) up-regulated and (b) down-regulated genes, overlapping in different compared groups (XLSX)

Supporting File 5. Cross-examination of (a) up-regulated and (b) down-regulated genes with the cancer genome atlas database for mutations, copy number variations, m-RNA expression and protein expression (XLSX)
